# Supplementary material for: Cul3-KLHL20 E3 ubiquitin ligase plays a key role in the arms race between HIV-1 Nef and host SERINC5 restriction
Source: Nat Commun. 2022 Apr 26;13:2242. doi: 10.1038/s41467-022-30026-y (PMC9042822; doi:10.1038/s41467-022-30026-y)
Supplement: Supplementary file 1 — Supplementary Information [file 41467_2022_30026_MOESM1_ESM.pdf]

Supplementary information

Cul3-KLHL20 E3 ubiquitin ligase plays a key role in the arms race between HIV-1 Nef and host SERINC5 restriction

Sunan Li[1,4], Rongrong Li[1,4], Iqbal Ahmad[1], Xiaomeng Liu[1], Silas F. Johnson[2], Liangliang Sun[3], and Yong-Hui Zheng[1,2]✉

[1]Harbin Veterinary Research Institute, CAAS-Michigan State University Joint Laboratory of Innate Immunity, State Key Laboratory of Veterinary Biotechnology, Chinese Academy of Agricultural Sciences, Harbin, China.
[2]Department of Microbiology and Molecular Genetics, Michigan State University, East Lansing, MI, USA.
[3]Department of Chemistry, Michigan State University, East Lansing, MI, USA.
[4]These authors contributed equally: Sunan Li, Rongrong Li.
✉email: zhengyo@msu.edu

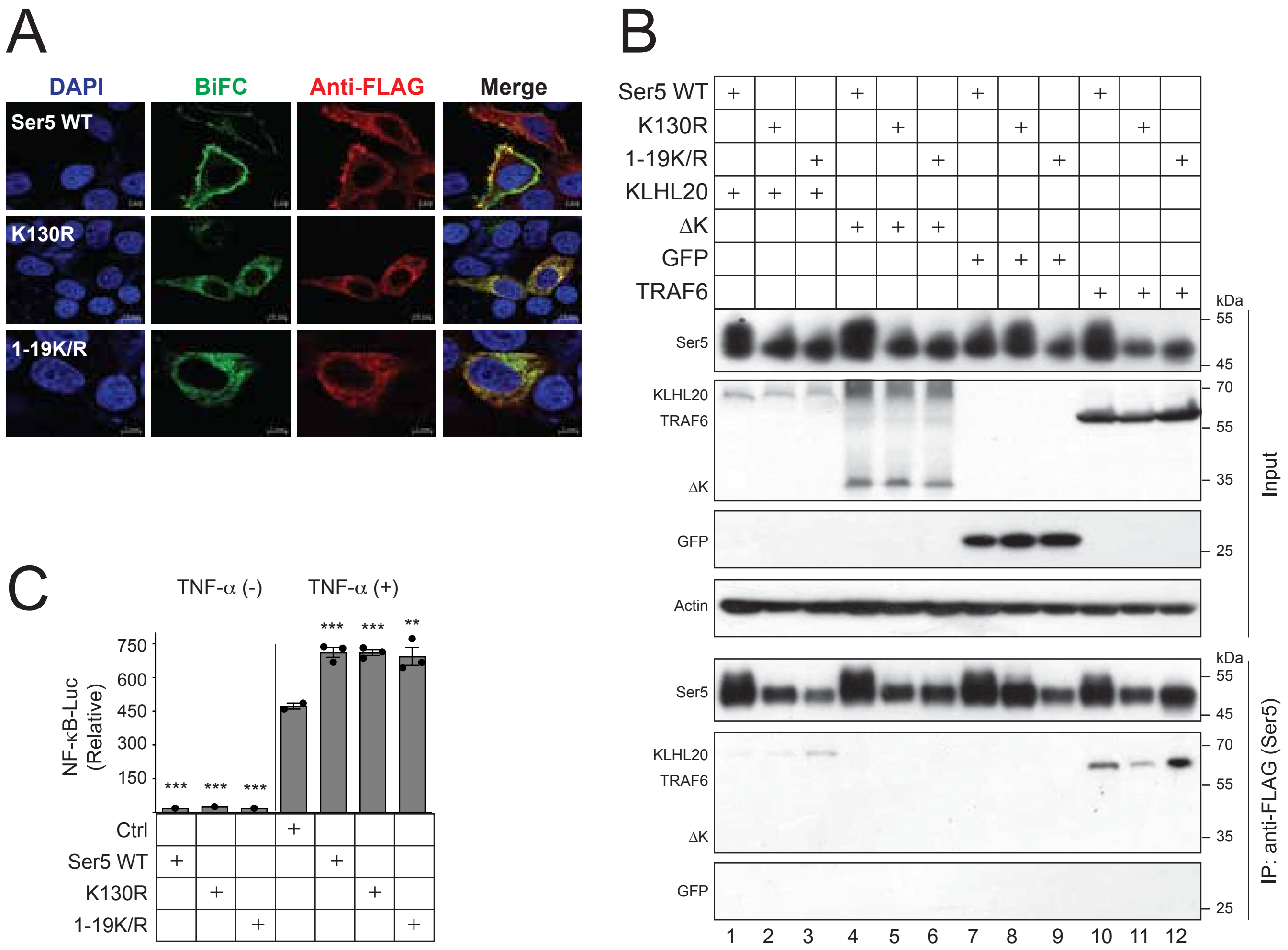

Supplementary Fig. 1. SERINC5 K130R mutant is functional and properly folded.

(A) Ser5-VN/Ser5-VC, K130R-VN/K130R-VC, and 1-19K/R-VN/1-19K/R-VC were expressed in pairs in HeLa cells. After staining with red-fluorescent anti-FLAG for the VN tag and DAPI for nuclei, cells were detected for expression of the green fluorescent BiFC signal and its colocalization with the red-fluorescent signal by confocal microscopy.

(B) FLAG-tagged Ser5 or its K130R and 1-19K/R mutants were expressed with HA-tagged KLHL20, its ΔK mutant, GFP, and TRAF6 in HEK293T cells. Cellular proteins were pulled down by anti-FLAG and analyzed by WB.

(C) Ser5 or its K130R and 1-19K/R mutants were expressed with a NF-κB promoter luciferase (Luc) reporter in HEK293T cells. Cells were treated with TNF-α (10 ng/mL) for 8 h. Forty-eight hours after transfection, the cells were lysed, and luciferase activities were measured. Results were shown as relative values, with that from cells remaining untreated with TNF-α as 1. Experiments were repeated twice, and similar results were obtained.

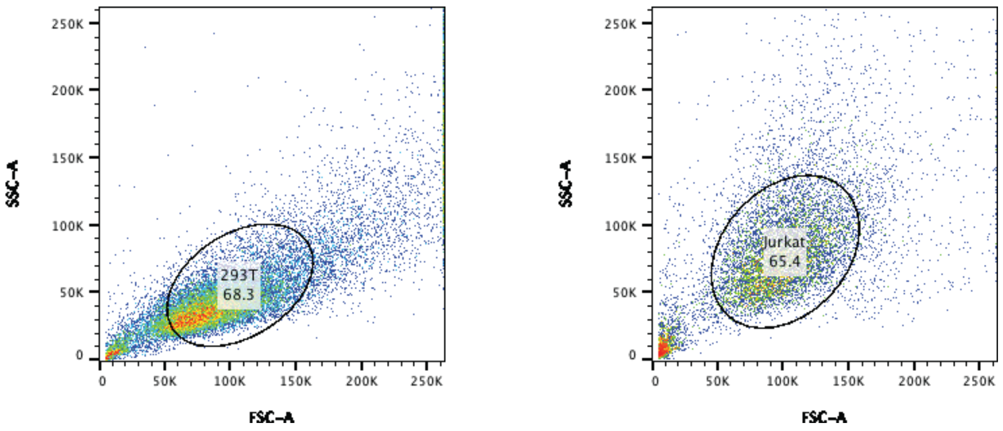

Supplementary Fig. 2. Gating strategy for HEK293T and Jurkat-TAg cells are shown, which was used to analyze Ser5 surface expression in Fig. 3B and Fig. 4A by flow cytometry.
